# Construction of a Drug Safety Assurance Information System Based on Clinical Genotyping

**DOI:** 10.5402/2012/982737

**Published:** 2011-11-29

**Authors:** John A. Springer, Nicholas V. Iannotti, Jon E. Sprague, Michael D. Kane

**Affiliations:** ^1^Department of Computer and Information Technology, Purdue University, West Lafayette, IN 47907, USA; ^2^Bindley Bioscience Center, Purdue University, IN 47907, USA; ^3^Raabe College of Pharmacy, Ohio Northern University, Ada, OH 45810, USA

## Abstract

To capitalize on the vast potential of patient genetic information to aid in assuring drug safety, a substantial effort is needed in both the training of healthcare professionals and the operational enablement of clinical environments. Our research aims to satisfy these needs through the development of a drug safety assurance information system (GeneScription) based on clinical genotyping that utilizes patient-specific genetic information to predict and prevent adverse drug responses. In this paper, we present the motivations for this work, the algorithms at the heart of GeneScription, and a discussion of our system and its uses. We also describe our efforts to validate GeneScription through its evaluation by practicing pharmacists and pharmacy professors and its repeated use in training pharmacists. The positive assessment of the GeneScription software tool by these domain experts provides strong validation of the importance, accuracy, and effectiveness of GeneScription.

## 1. Introduction

The utilization of a clinical patient's genetic data to aid diagnostic and prognostic healthcare represents the ultimate achievement of fifty years of genomic research. However, some operational, ethical, and educational challenges hinder the implementation of a societal-scale clinical genotyping system even though the technologies to carry out clinical genotyping do exist. To overcome these hurdles, we have developed a data management system (GeneScription) that utilizes patient-specific genotyping to predict and prevent adverse drug responses and thus supports the prescription drug process from physician to pharmacist to consumer. The system uses specific allelic variables associated with drug metabolism, as well as other common laboratory tests, to identify patients that are predisposed to an adverse drug reaction, and make recommendations as to the best course of action for a particular drug and patient. The GeneScription system represents the first software system of its kind in that it supports a key component of healthcare (prescription drugs) that is not ethically constrained by the prediction and prognosis of serious disease through patient-specific DNA variance and is therefore acceptable to the healthcare consumer.

Moreover, since most practicing physicians and pharmacists were trained long before the utilization of human genomic information was seriously considered as a component of healthcare, educating these healthcare professionals is paramount to the future clinical genotyping adoption. To address this need, GeneScription also provides in depth training for the user (physician or pharmacist) to better understand the link between DNA (genes), drug metabolism (enzymes), and the risk of adverse drug responses within prescription medicine. The system includes (1) a population of mock patients having in many cases a specific genetic predisposition for an adverse drug response and (2) all drugs approved by the United States Food and Drug Administration.

In the following, we first provide motivation for the development of the GeneScription system and describe others' related work. We then proceed to an explanation of the algorithms upon which we base GeneScription and a description of the results related to our development efforts. We next have a discussion of our research before concluding with a summary of this paper and an exploration of future work.

## 2. Motivation

The Institute of Medicine estimates that 7,000 deaths occur annually due to adverse drug reactions (ADRs) [1]. Other studies have suggested that in the hospital setting 6.7% or over 2 million hospitalized patients experience ADRs with over 100,000 of those patients succumbing to ADRs [2], making ADRs the 4th leading cause of death in the United States. In addition, data suggest that serious adverse drug reaction events have doubled in the last decade [3]. Based on this evidence, ADRs are one of today's leading, preventable public health issues, and the number of ADR events will likely continue to grow in the future.

ADRs associated with the therapeutic treatment of disease in many cases are coupled with elevations in plasma drug concentrations. Alterations in drug metabolism directly influence plasma concentrations. For example, CYP2D6 and CYP2C9 mutations have been associated with elevations in concentrations in paroxetine [4] and warfarin [5] levels, respectively. Therefore, increasing the accessibility and utility of genomic screening for CYP polymorphisms (drug metabolism enzymes) will reduce ADRs. In fact, this utility is already widely recognized in that some drug labels now provide explicit directions that the drug's use is contraindicated in cases involving the presence of variant alleles, such as thioridazine and CYP2D6 poor metabolizers [6].

In addition, response to drug therapy varies markedly across therapeutic areas. For example, the estimated response rate to the selective serotonin reuptake inhibitors (SSRIs) used in the treatment of depression is 60% [7]. The resistance to the antiplatelet drug clopidogrel has been estimated to be up to 30% [8]. Clopidogrel is a prodrug that requires CYP2C19 bioactivation [9]. Pharmacogenomic screening can both reduce the rate of ADRs and also enhance overall therapeutic response to drug therapy by identifying patients deficient in prodrug bioactivation processes.

Despite the clear benefits, many factors have contributed to obstacles that limit the translation of genomic data to routine use in patient care. Concerns over privacy, security, and ethical issues are just a few of the issues that have limited this translation from “bench to bedside.” To address these concerns in the GeneScription system, we target known single nucleotide polymorphisms (SNPs) in P450 metabolizing enzymes, and GeneScription stores only the relevant information for these SNPs; we do not collect any other information related to genomic anomalies.

As we discussed in Section 1, another limiting factor is related to education. It is crucial to educate healthcare professionals within the realm of clinical genomics to facilitate the future of this powerful approach to improve medical outcomes [10–12]; this too is being addressed by the GeneScription project.

## 3. Related Work

Having established the significance of our research, we now turn to a discussion of related work. In this section, we briefly address system-related considerations as well as the state-of-the-art with regard to current systems similar to GeneScription.

### 3.1. System Considerations

The implementation of a societal-scale personalized medicine system—one of the ultimate goals of the application of pharmacogenomics to current healthcare practices—must first satisfy several preexisting conditions and requirements before widespread adoption is possible. As provided in [12], gaps exist in the several areas that must be addressed before a societal-scale personalized medicine system; these areas include the availability of cost-effective high-throughput DNA analysis technology, the establishment of point-of-care utilization of genomics, and the establishment of translational research linkages between patient genotype data and healthcare. Each of these aspects has an effect on the characteristics that a societal-scale personalized medicine system will exhibit; we refer interested readers to [12].

### 3.2. Current Systems

The application of technology to healthcare has resulted in the development of clinical decision support systems (CDSS), many of which are currently in use today. Such systems have been shown to prevent errors, improve quality of care, reduce costs, and save personnel time. The effectiveness of such systems has been quantified, with one study of nearly 100 implementations citing an increase in performance of practitioners in 64% of the instances, an increase in performance in 40% of diagnostic systems, 76% of remainder systems, 62% of disease management systems, and 66% of drug-dosing or prescribing systems [13]. However, despite this and other studies implicating the potential of CDSSs to improve the quality of care, such systems have not gained widespread use outside of large academic medical centers and integrated delivery systems [14]. An extensive review of the known CDSSs is beyond the scope of this paper; as described in [15], notable systems include the Regenstrief Medical Record System [16], Epic Systems [17], McKesson Horizon Expert Orders [18], Cerner Millennium [19], and VistA from the United States Veterans Administration [20]. In addition, a number of standards-based approaches exist, including Arden Syntax [21] and Guideline Interchange Format (GLIF) [22], as do service models such as the Shareable Active Guideline Environment (SAGE) [23] and the System for Evidence-Based Advice through Simultaneous Transaction with an Intelligent Agent across a Network (SEBASTIAN) [24]. None of the known clinical decision support systems currently integrate drug safety assurance with clinical genotyping in the manner in which GeneScription does.

## 4. Methods

We now focus on the methods that we use in the development of the GeneScription software and present the scientific basis and algorithmic processes that we utilize in GeneScription. The approach that we employ is largely a result of first applying a categorization to the aspects of pharmacotherapy that are most relevant to a particular patient. Then, within those categories, we leverage the most precise known and relevant pharmacokinetic (PK) equations to provide guidance to the user.

For prodrug conversion conflicts, if data is available that quantifies the extent of conversion to the parent compound, a multiplier is calculated and used in dosage computation. This multiplier is typically determined from either the average dose needed by a patient harboring the variant allele or the extent to which enzymatic activity is altered. For example, as given by Marinaki et al. [25], for the conversion of the prodrug azathioprine to the active metabolite 6-mercaptopurine. Otherwise, such as in the case of patients harboring CYP2D6 variant alleles and the conversion from codeine to morphine, the system cannot yet reliably provide accurate pharmacokinetic calculations [26]. In this case, the user is alerted that conversion from the prodrug to the target drug may be altered and the target drug's mechanism of action as a result could be affected.

If a variant results in the production of a null enzyme, this is treated in a similar way. If information exists about the extent to which metabolism is affected, then that information is used to calculate an appropriate dosage. This can be accomplished using a multiplier as described previously for prodrug conversion, such as in the calculation of an appropriate clopidogrel dosage in patients harboring the CYP2C19∗2 allele [27]. Otherwise, the user is warned of the risk of altered metabolism as this could result in drug toxicity, or in the case of a prodrug, the possibility that normal dosing may not result in therapeutic blood plasma levels due to decreased bioconversion.

In the case that the patient harbors variants that do not produce null enzymes and the drug prescribed is not a prodrug, we employ the most accurate in vivo quantification of the influence of metabolic variants present in the patient's genotyping test results. From a high-level, this can be described as the dynamic composition of a personalized pharmacogenomic algorithm [28]. Sequence variation at the allelic level as defined by Den Dunnen and Antonarakis [29] can be specified at the nucleotide or amino acid sequence (protein) levels. For example, in the case of warfarin metabolism and the variant CYP2C9∗2, there exists clinical metabolic data at the suballele level (∗2A, ∗2B, and ∗2C) that is used in lieu of more general CYP2C9∗2 data [30].

If no data is available at the allelic level, group studies of patients exhibiting variant metabolic function are then used. Depending on the variant, these are typically classified as “poor metabolizer” (PM) or “ultra metabolizer” (UM) studies. These studies typically either provide multipliers that describe the average changes noted in peak plasma concentrations, quantify the necessary differences in dosage, or may provide alternative values for pharmacokinetic calculations, such as decreased clearance values or increased half-life [31]. If no match is made, then the system uses a 33% or 66% increase in drug half-life and expected peak plasma concentration to project and estimate drug metabolism. That is, if the patient harbors a single nucleotide polymorphism (SNP) and is heterozygotic (one variant and one normal/wild-type copy is present), the 33% rule is applied; if the patient is homozygotic (both copies are variants), then the 66% rule is applied.

In every appropriate situation, the system visually alerts the user to the presence of variant alleles. In Algorithms 1, 2, and 3, we present the GeneDrugMatch, Graph, and CreateDoseCurve algorithms that implement the aforementioned approach. Of particular note are (1) and (2) that we use in calculating the peak plasma concentration, as found in [31]. In (1), we base our calculation on a single compartment model and a first-order pharmacokinetic process [31]; as for (2), we use the equation therein in our calculation of the peak plasma concentration found in (1). A few additional comments are necessary. Specifically, the peak plasma concentration (*C*_*p*_^0^ or *C*_max_) is calculated by extrapolation to *t* = 0. However, as computation is not possible using *t* = 0, we use *t* = 0.00000001 instead. Secondly, after calculating the peak plasma concentration, it is plotted according to the provided value for the drug's time to peak concentration.

Calculation of drug plasma concentration

(1) $$\begin{array}{c} C_{p}=\frac{F\times D}{V_{d}\times W}\times \left(e^{-kt}\right) \end{array},$$

where *C*_*p*_ = drug plasma concentration (*μ*g/mL), *F* = bioavailability (%), *D* = drug dosage (mg), *V*_*d*_ = volume of distribution (L/g), *W* = patient weight (kg), *k* = rate constant for elimination (hr^−1^), and *t* = time (hr).

Fraction of drug removed from central compartment per unit of time

(2) $$\begin{array}{c} k=\frac{0.693}{t_{1/2}},\; \end{array}$$

where *t*_1/2_ = drug  half-life  (hr).

**Figure 1:**
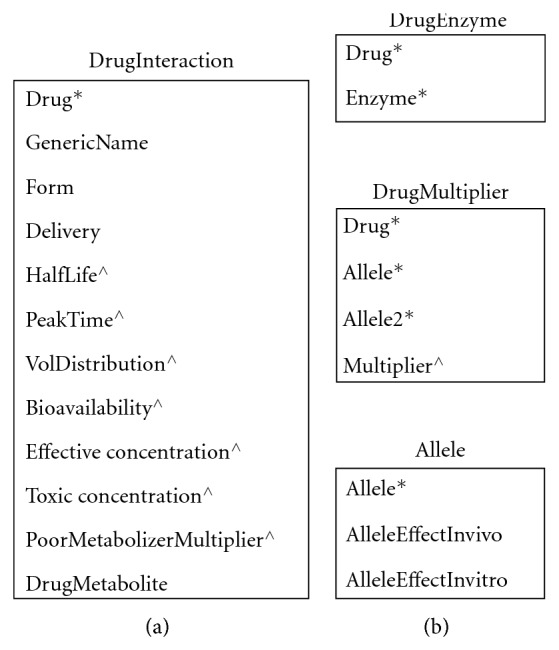
Tables (∗ denotes an identifier.  ^∧^ denotes float).

**Figure 2:**
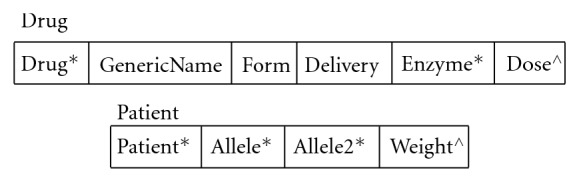
Data structures (∗ denotes an identifier.  ^∧^ denotes float).

In addition, we refer to a series of tables and data structures in our algorithms, and we provide the abbreviated schemas for the tables and the components of the data structures in Figures 1 and 2, respectively. GeneScription contains data for all drugs approved by the Food and Drug Administration [32] and currently uses fictitious patients to mitigate privacy concerns. In addition, GeneScription includes hyperlinks to the studies that determine the multiplier and other attribute values employed by GeneScription. We collect and validate the study data that we place in GeneScription by leveraging our project's domain experts to evaluate and approve the use of said data; we elaborate on data collection and validation later in this paper.

Note that Figure 1 provides only a subset of the tables' columns; moreover, the tables in Figure 1 are a small subset of the tables found in the implementation of the GeneScription system. In these tables and records, the data type for a column is a string unless marked with an ∗ denoting an integer data type serving as an identifier or a  ^∧^ denoting a float data type. Additionally, please notice that the DrugInteraction record to which we refer in the GeneDrugMatch algorithm (Algorithm 1) replicates the structure of the DrugInteraction table in Figure 1, and hence we do not provide this repetitive information in Figure 2.

As for software development methods, we used a standard lifecycle and accepted best practices such as version control and code reviews. In particular, given the always evolving nature of pharmacogenomics, we needed agile development methods that enabled a feedback loop in which the domain experts provided frequent feedback to the development team that the development team then in turn incorporated into the software.

Subsequent to the inception of the concept, we used rigorous requirements discovery/prototyping cycles that enabled us to quickly collect, verify, and refine requirements with our team's domain expertise. These cycles were short and frequent, and because of our approach, it was more important to initially define and collect requirements than to optimize at this point in the development process. Nevertheless, scalability and optimization were not ignored at this juncture; instead, final decisions on issues affecting scalability and optimization were postponed as long as possible.

In addition, prototyping did require us to develop components that were later not used; this allowed us to speed requirements discovery but did not directly contribute to operational software. However, since it was paramount that we quickly integrate feedback from our team's domain experts and thereby tighten our cycles, we accepted this tradeoff. As for development tools, Visual Studio.NET Framework 2.0 and Microsoft Access were chosen for the prototype as they provided the quickest avenue to a functional prototype using technology readily available.

The discovered requirements were substantial and included typical needs such the ability to store, relate, and allow the user to interact with and display large amounts of information containing complex relationships in a way that does not burden the user or overload them with information that is not needed. In addition, we uncovered requirements that are very particular to the area, such as an understanding of the factors that determine the relationship between the prescribed drug dosage and drug effect.

After requirements discovery and prototyping, we proceeded to validate the requirements by engaging focus groups that consisted of fifth and sixth year pharmacy school students, pharmacy school educators, and practicing pharmacists. The results from these focus groups were then incorporated into the final, nearly “production-ready” prototype, and this final prototype served as the input into our efforts in the development of an enterprise-scale system. The resulting enterprise-scale system serves as the basis for both the educational system as well as the societal-scale operational system. We developed the enterprise-scale version of GeneScription using Microsoft Visual Studio 2008 and Expression Blend 3, and it is a Silverlight 3 application that uses Entity Framework and LINQ,.NET RIA services, Domain services, and the Windows Communication Foundation (WCF) on the client side as well as ASP.NET 3.5, Internet Information Services 7, SQL Server 2008, and Windows Server 2008 Datacenter Edition on the server side.

## 5. Results

We now turn from a description of our methods to an examination of our results. Since the utilization of clinical genotyping for the purposes of drug safety assurance is not routinely practiced in healthcare for reasons that include the current absence of cost-effective high-throughput DNA analysis technology (as mentioned in the Section 3), the validation of the utility of the GeneScription software in a clinical environment is currently not feasible. However, the software has been evaluated by practicing pharmacists and pharmacy professors, and repeatedly used for training pharmacists in the drug dispensing laboratory in the School of Pharmacy at Ohio Northern University (ONU). Results from a pre- and postsurvey of participants in the clinical laboratory training environment at ONU where GeneScription was utilized for training in personalized medicine showed that all participants “strongly agreed” that genomic data can be used to determine the optimal dose of a drug and demonstrated a significantly increased willingness to submit DNA data for genetic profiling as well as an increased understanding of the manner in which DNA analysis is completed. Since these participants have expert knowledge in pharmacology and pharmaceutics, their positive assessment of this software tool provides strong validation in the absence of real-world clinical healthcare opportunities for software assessment.

**Figure 3:**
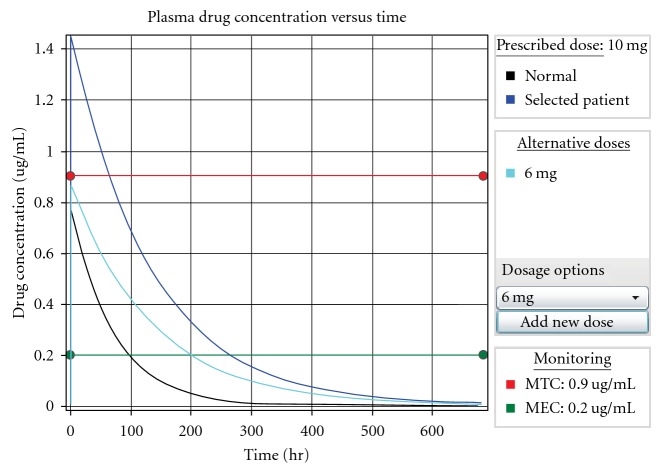
Dose curves with minimum effective and toxic concentrations.

In Figure 3, we illustrate a few important aspects of the enterprise-scale version of the GeneScription system, which is available in its entirety at http://www.genescription.com/. Before arriving at the information presented in Figure 3, the user would have selected a patient and then a drug, and if warranted by the patient's genetic profile in concert with the selected drug, GeneScription would then detect an interaction and warn the user. Next, the user would have the opportunity to view the current dose curve and select an alternate dose. After selecting an alternative, the user would then see the dose curve that is associated with the alternate dose. Figure 3 depicts the result of this step and includes a user-selected dosing alternative (in the figure, 6 mg), the “normal” (i.e., a patient not harboring a variant allele) and selected patient dose curves, and the minimum effective (denoted as MEC in the figure) and toxic (denoted as MTC in the figure) concentrations. Of primary importance in the alternative, normal, and selected patient curves are the peak plasma concentrations denoted by the respective curves' highest *Y* coordinates or “peaks.” Note that in the alternative and normal cases, their peaks sit below the minimum toxic concentration level while the selected patient's peak stands well above the minimum toxic concentration level; this is the condition that would have triggered the warning to the user. Although not shown in Figure 3, GeneScription would also present (1) the patient's clinical (such as weight) and genotypic data and (2) the drug's pharmacokinetic data including links to pertinent data on PharmGKB [33].

## 6. Discussion

Having reported on our construction of the GeneScription system, it is important to reiterate that GeneScription is based on a “hierarchical” approach in that for each case (i.e., each patient and drug combination under scrutiny), we apply the most selective method in our hierarchy for determining the extent of the “drug-gene” interaction and only use a less selective method if a more selective one is not appropriate. In essence, GeneScription evolves as the body of knowledge concerning SNPs evolves. In fact, new research emerged during the course of our implementation efforts, and GeneScription quickly incorporated this new knowledge after careful vetting by our team of experts.

In addition to the need to adapt rapidly to an ever evolving body of knowledge, the GeneScription system must deal with a host of nontechnical considerations if it is to achieve the widespread adoption to which it aspires. We mentioned some of these considerations in Section 3, and in this section, we elaborate briefly on a few of these considerations, beginning with the ethics that surround the use of clinical genotyping in drug safety assurance. The ethical concerns to genotyping in the clinic, which are also applicable to electronic health records in general, include privacy and security. The benefits of incorporating genotyping (genetic information) in therapeutics and medicine are questioned when the risk of “information abuse” is considered. For example, a patient may be unwilling to utilize the benefits of genotyping if they fear that their employer and/or insurance provider can utilize the same information to (accurately or inaccurately) predict the patient's future health status. This dilemma involves both societal and genetic components. At the genetic level, the validity of extrapolative health assessment based solely on genotypic data has not been broadly established and is limited to a few known genetic diseases. Yet, it should be noted that the risk of adverse drug response based on known SNPs in drug metabolism enzymes has been established [12], and represents a benefit of clinical genotyping that could be realized in the short-term. At the societal level, studies such as [34, 35] indicate that the public has concerns related to the capacity of clinical genotyping to possibly introduce racial or class inequalities in access to treatment. For a fuller treatment of the ethical concerns surrounding clinical genotyping, please review [36] in [37].

Beyond ethical concerns, the education of current healthcare professionals and patients in genomics and genetics is essential for wide adoption of clinical genotyping in drug safety assurance. As with the application of any new technology, especially those affecting such a critical service as healthcare, in an actual commercial application there will be hesitation toward initial acceptance. Success of the GeneScription system rests firmly on the availability of information, training, and education for all potential stakeholders. Patients need to understand the manner in which we use their genomic data, and as this system requires the input of data inherently of a personal nature, patients must trust the system. As we discussed earlier, in the use of genomic data as applied to drug safety, the indication that a patient would be predisposed to an adverse drug effect does not implicate an association with other health risks. This creates a situation where all interested parties benefit from this knowledge and reduces the level of understanding and education needed to make informed decisions [12]. Hence, the specific application of clinical genotyping toward drug safety represents an optimal environment for initial consideration and implementation of personalized medicine. Before the advent of clinical genotyping, numerous studies indicate that the close involvement of the patient in drug therapy can greatly affect the success of the outcome ([38–40] among many). Ultimately, this must be repeated in the context of clinical genotyping for drug safety assurance to enable the degree of adoption warranted by the promise of the GeneScription system. GeneScription, though, is in a unique position in that it not only functions as an operational system but also is intended to assist stakeholders in understanding the application of clinical genotyping to drug safety assurance, and hence the GeneScription system can play an extremely active role in its own acceptance.

Another obstacle relates to data collection and validation as noted in the previous section. Lack of consensus on how best to manage patients with SNPs altering drug metabolism or drug targets further hinders the adaptation of clinical genotyping in the everyday practice of medicine. We propose the formation of the Pharmacogenetic National Committee (PNC) on the use of genetic information for the pharmacologic management of disease which would meet annually to establish pharmacogenetic dosing guidelines. A critical mass of information accumulated on pharmacogenetic dosing has generated the need for consensus dosing guidelines. The formation of the PNC is predicated on (1) the need for a clear and concise guideline that would be useful to clinicians, (2) the publication of numerous observational studies and clinical trials relating to pharmacogenetic dosing, and (3) the clear recognition that pharmacogenetic dosing is not being utilized to its maximum potential. Systems like GeneScription will assist in building the consensus needed to make clinical genotyping a major component of everyday patient care.

## 7. Conclusion

In this paper, we provided motivation for the development of the GeneScription system and discussed others' related work, including an overview of related clinical decision support systems. We also explained in detail the algorithms that are at the core of the GeneScription system and described our efforts throughout the implementation process. We then reported our results from the evaluation of GeneScription by practicing pharmacists and pharmacy professors and the feedback provided by the repeated use of GeneScription for training pharmacists in the drug dispensing laboratory in the School of Pharmacy at Ohio Northern University. Additionally, we included a discussion of aspects of GeneScription that are pertinent to its successful use and adoption; this section contained details concerning the ethical and educational considerations related to GeneScription's adoption.

GeneScription continues to evolve to meet the accuracy requirements dictated by the ever changing landscape of SNP research. We are also in the process of extending GeneScription to mobile device platforms such as the Apple iPhone and developing some additional education tools to assist in advancing the adoption of GeneScription in particular and more generally clinical genotyping for drug safety assurance.

## Figures and Tables

**Algorithm 1:**
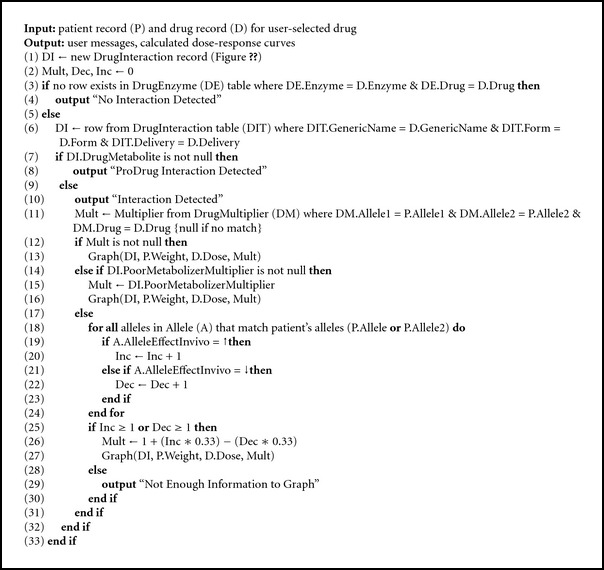
GeneDrugMatch algorithm.

**Algorithm 2:**
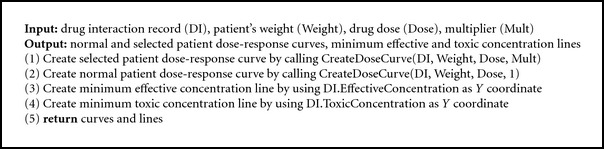
Graph algorithm.

**Algorithm 3:**
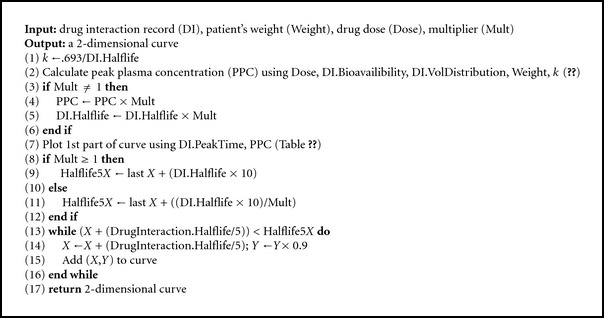
CreateDoseCurve algorithm (*X* and *Y* refer to coordinates in curve).

**Table 1:** Points in first part of dose curve.

Sequence	*X*	*Y*
1	.05 PT	.005 PPC
2	.1 PT	.12 PPC
3	.15 PT	.23 PPC
4	.2 PT	.36 PPC
5	.25 PT	.50 PPC
6	.3 PT	.66 PPC
7	.35 PT	.75 PPC
8	.4 PT	.82 PPC
9	.45 PT	.87 PPC
10	.5 PT	.90 PPC
11	.55 PT	.92 PPC
12	.6 PT	.94 PPC
13	.65 PT	.96 PPC
14	.70 PT	.97 PPC
15	.75 PT	.98 PPC
16	.80 PT	.99 PPC
17	.85 PT	.993 PPC
18	.90 PT	.996 PPC
19	.95 PT	.998 PPC
20	1.0 PT	1.0 PPC
21	1.05 PT	.998 PPC

PT = % of peak time

PPC = % of peak plasma concentration.

## References

[1] Institute of Medicine Committee on Quality of Health Care in America (2000). *To Err Is Human: Building a Safer Health System*.

[2] Lazarou J., Pomeranz B. H., Corey P. N. (1998). Incidence of adverse drug reactions in hospitalized patients: a meta- analysis of prospective studies. *Journal of the American Medical Association*.

[3] Moore T. J., Cohen M. R., Furberg C. D. (2007). Serious adverse drug events reported to the food and drug administration, 1998–2005. *Archives of Internal Medicine*.

[4] Sawamura K., Suzuki Y., Someya T. (2004). Effects of dosage and CYP2D6-mutated allele on plasma concentration of paroxetine. *European Journal of Clinical Pharmacology*.

[5] Aithal G. P., Day C. P., Kesteven P. J. L., Daly A. K. (1999). Association of polymorphisms in the cytochrome P450 CYP2C9 with warfarin dose requirement and risk of bleeding complications. *Lancet*.

[6] Avigan M. I. (2009). Pharmacogenomic biomarkers of susceptibility to adverse drug reactions: just around the corner or pie in the sky?. *Personalized Medicine*.

[7] Doris A., Ebmeier K., Shajahan P. (1999). Depressive illness. *Lancet*.

[8] Nguyen T. A., Diodati J. G., Pharand C. (2005). Resistance to clopidogrel: a review of the evidence. *Journal of the American College of Cardiology*.

[9] Mega J. L., Close S. L., Wiviott S. D. (2009). Cytochrome P-450 polymorphisms and response to clopidogrel. *New England Journal of Medicine*.

[10] Aspinall M. G., Hamermesh R. G. (2007). Realizing the promise of personalized medicine. *Harvard Business Review*.

[11] Chung W. K. (2007). Implementation of genetics to personalize medicine. *Gender Medicine*.

[12] Kane M. D., Springer J. A., Sprague J. E. (2008). Drug safety assurance through clinical genotyping: near-term considerations for a system-wide implementation of personalized medicine. *Personalized Medicine*.

[13] Garg A. X., Adhikari N. K. J., McDonald H. (2005). Effects of computerized clinical decision support systems on practitioner performance and patient outcomes: a systematic review. *Journal of the American Medical Association*.

[14] Wright A., Sittig D. F. (2008). A framework and model for evaluating clinical decision support architectures. *Journal of Biomedical Informatics*.

[15] Dean F. S., Linas S., James D. C. (2010). The state of the art in clinical knowledge management: an inventory of tools and techniques. *International Journal of Medical Informatics*.

[16] Overhage J., McDonald C. J., Suico J. G. The regenstrief medical record system 2000: expanding the breadth and depth of a community wide EMR.

[17] Chin H. L., Krall M. A. (1998). Successful implementation of a comprehensive computer-based patient record system in Kaiser Permanente Northwest: strategy and experience. *Effective Clinical Practice*.

[18] Jensen J. The effects of computerized provider order entry on medication turn-around time: a time-to-first dose study at the providence Portland Medical Center.

[19] Lechleitner G., Pfeiffer K. P., Wilhelmy I., Ball M. (2003). Cerner millennium: the innsbruck experience. *Methods of Information in Medicine*.

[20] Naditz A. (2008). Telemedicine at the VA: VistA, MyHealtheVet, and other VA programs. *Telemedicine and e-Health*.

[21] Hripcsak G. (1991). Arden Syntax for medical logic modules. *M.D. Computing*.

[22] Wang D., Peleg M., Tu S. W. (2004). Design and implementation of the GLIF3 guideline execution engine. *Journal of Biomedical Informatics*.

[23] Tu S. W., Campbell J. R., Glasgow J. (2007). The SAGE guideline model: achievements and overview. *Journal of the American Medical Informatics Association*.

[24] Kawamoto K., Lobach D. F. Design, implementation, use, and preliminary evaluation of Sebastian, a standards-based web service for clinical decision support.

[25] Marinaki A. M., Ansari A., Duley J. A. (2004). Adverse drug reactions to azathioptine therapy are associated with polymorphism in the gene encoding inosine triphosphate pyrophosphatase (ITPase). *Pharmacogenetics*.

[26] Sheffield L. J., Phillimore H. (2009). Clinical use of pharmacogenomic tests in 2009. *The Clinical Biochemistry Reviews*.

[27] Varenhorst C., James S., Erlinge D. (2009). Genetic variation of CYP2C19 affects both pharmacokinetic and pharmacodynamic responses to clopidogrel but not prasugrel in aspirin-treated patients with coronary artery disease. *European Heart Journal*.

[28] Consortium T. I. W. P. (2009). Estimation of the warfarin dose with clinical and pharmacogenetic data. *The New England Journal of Medicine*.

[29] Den Dunnen J. T., Antonarakis S. E. (2000). Mutation nomenclature extensions and suggestions to describe complex mutations: a discussion. *Human Mutation*.

[30] King B. P., Khan T. I., Aithal G. P., Kamali F., Daly A. K. (2004). Upstream and coding region CYP2C9 polymorphisms: correlation with warfarin dose and metabolism. *Pharmacogenetics*.

[31] Thummel K. E., Shen D. D., Isoherranen N., Smith H. E. (2006). Design and optimization of dosage regimens: pharmacokinetic data. *Goodman & Gilman’s The Pharmacological Basis of Therapeutics*.

[32] Food and Drug Administration (2010). *Orange Book: Approved Drug Products with Therapeutic Equivalence Evaluations*.

[33] Klein T. E., Chang J. T., Cho M. K. (2001). Integrating genotype and phenotype information: an overview of the PharmGKB project. *Pharmacogenomics Journal*.

[34] Almarsdóttir A. B., Björnsdóttir I., Traulsen J. M. (2005). A lay prescription for tailor-made drugs - Focus group reflections on pharmacogenomics. *Health Policy*.

[35] Bevan J. L., Lynch J. A., Dubriwny T. N. (2003). Informed lay preferences for delivery of racially varied pharmacogenomics. *Genetics in Medicine*.

[36] Springer J. A., Beever J., Morar N., Sprague J. E., Kane M. D., Dark M. J. (2011). Ethics, privacy and the future of genetic information in healthcare information assurance and security. *Information Assurance and Security Ethics in Complex Systems: Interdisciplinary Perspectives*.

[37] Dark M. J. (2011). *Information Assurance and Security Ethics in Complex Systems: Interdisciplinary Perspectives*.

[38] Lobas N. H., Lepinski P. W., Abramowitz P. W. (1992). Effects of pharmaceutical care on medication cost and quality of patient care in an ambulatory-care clinic. *American Journal of Hospital Pharmacy*.

[39] Pauley T. R., Magee M. J., Cury J. D. (1995). Pharmacist-managed, physician-directed asthma management program reduces emergency department visits. *Annals of Pharmacotherapy*.

[40] Doucette W. R., Andersen T. N. (2005). Practitioner activities in patient education and drug therapy monitoring for community dwelling elderly patients. *Patient Education and Counseling*.

